# Palmitic Acid Promotes Virus Replication in Fish Cell by Modulating Autophagy Flux and TBK1-IRF3/7 Pathway

**DOI:** 10.3389/fimmu.2020.01764

**Published:** 2020-08-05

**Authors:** Yepin Yu, Chen Li, Jiaxin Liu, Fengyi Zhu, Shina Wei, Youhua Huang, Xiaohong Huang, Qiwei Qin

**Affiliations:** ^1^Joint Laboratory of Guangdong Province and Hong Kong Region on Marine Bioresource Conservation and Exploitation, College of Marine Sciences, South China Agricultural University, Guangzhou, China; ^2^Guangdong Laboratory for Lingnan Modern Agriculture, Guangzhou, China; ^3^Laboratory for Marine Biology and Biotechnology, Qingdao National Laboratory for Marine Science and Technology, Qingdao, China

**Keywords:** palmitic acid, grouper, SGIV, replication, interferon, autophagy flux

## Abstract

Palmitic acid is the most common saturated fatty acid in animals, plants, and microorganisms. Studies highlighted that palmitic acid plays a significant role in diverse cellular processes and viral infections. Accumulation of palmitic acid was observed in fish cells (grouper spleen, GS) infected with Singapore grouper iridovirus (SGIV). The fluctuated content levels after viral infection suggested that palmitic acid was functional in virus-cell interactions. In order to investigate the roles of palmitic acid in SGIV infection, the effects of palmitic acid on SGIV induced cytopathic effect, expression levels of viral genes, viral proteins, as well as virus production were evaluated. The infection and replication of SGIV were increased after exogenous addition of palmitic acid but suppressed after knockdown of fatty acid synthase (FASN), of which the primary function was to catalyze palmitate synthesis. Besides, the promotion of virus replication was associated with the down-regulating of interferon-related molecules, and the reduction of IFN1 and ISRE promotor activities by palmitic acid. We also discovered that palmitic acid restricted TBK1, but not MDA5-induced interferon immune responses. On the other hand, palmitic acid decreased autophagy flux in GS cells via suppressing autophagic degradation, and subsequently enhanced viral replication. Together, our findings indicate that palmitic acid is not only a negative regulator of TBK1-IRF3/7 pathway, but also a suppressor of autophagic flux. Finally, palmitic acid promotes the replication of SGIV in fish cells.

## Introduction

Lipids, the major components of biological cell membranes, play essential roles in intracellular signaling, and act as the precursors for ligands' binding to nuclear receptors (1–3). Lipids are involved in various cellular processes, such as autophagy (4, 5), a conserved biological pathway that delivers cytoplasmic components to lysosomes and maintains the balance between synthesis, degradation, and recycling of cellular components (4, 6). On the other hand, different viruses demonstrate differential manipulation of cellular lipid metabolism (7–9). Specifically, the developments of specific cellular microenvironments created by viruses through specialized virus-induced organelle-like structures within infected cells, benefit viral replication (10–12). Moreover, in some circumstances, lipids could be utilized by viruses as signaling molecules (13, 14). Palmitic acid, known as the most common fatty acid (FA) found in animals, plants, and microorganisms, has been reported to be associated with autophagy modulation and inflammatory responses in mammal cells (15, 16). Recent research on zebrafish suggests palmitic acid processes antiviral activity via its inhibition of autophagic flux (17). Another report revealed that live *Edwardsiella tarda* vaccine promoted biosynthesis of palmitic acid and then increased the IL-8 expression in zebrafish, and subsequently contributed to the resistance against *E. tarda* infection (18). Moreover, palmitic acid has an inhibitory effect on IFN-based anti-Hepatitis C Virus (HCV) therapy (19). Palmitic acid plays a significant role in cell autophagy and pathogen-host interactions.

Orange-spotted grouper, *Epinephelus coioides*, is one of the commercially important farmed fishes in China and Southeast Asian countries. However, outbreaks of viral and bacterial diseases always cause massive economic losses and affect the development of grouper aquaculture (20–23). Among these pathogens, Singapore grouper iridovirus (SGIV) was identified for causing high fish mortality at different stages in grouper aquaculture (20, 22). Efforts have been made in discovering the infection mechanisms of SGIV by characterizing the functions of host immune-related genes as well as crucial viral virulence genes (21, 24–31). Additionally, findings of the role of lipids on modulating cell immunity have been advanced. For example, grouper 25-hydroxycholesterol (Ec-25HC) indicated antiviral activity against SGIV (32). Furthermore, the characterization of cellular lipid metabolism revealed the increased palmitic acid level in SGIV infected cells (unpublished data). Although considerable progress had been made in molecular analysis of viral infection or host antiviral strategies, the functions of FAs, especially palmitic acid, in fish virus infection, remained mostly unclear. The investigation of palmitic acid would benefit the understanding of SGIV pathogenesis.

In the present study, the effects of palmitic acid over-loading on host cell viability, virus replication, interferon-related gene expression, and cell autophagy were characterized. SGIV infection led to the accumulation of palmitic acid in GS cells. By suppressing cell autophagic flux and the TBK1-IRF3/7 signaling pathway, palmitic acid finally facilitated the replication of SGIV. Thus, we speculated that SGIV utilized palmitic acid in immune evasion processes. Our results provided new insights into the biological activity of palmitic acid and increased the understanding of SGIV pathogenesis as well.

## Materials and Methods

### Cell Lines and Virus

Grouper spleen (GS) cell line from grouper, *Epinephelus akaara*, was established in our laboratory (33). GS cells were cultured in Leibovitz's L-15 medium containing 10% fetal bovine serum (FBS, Gibco) at 25°C (33). Singapore grouper iridovirus (SGIV, strain A3/12/98 PPD) was propagated in GS cells and stored at −80°C until use (34).

### Cell Treatment

Cellular toxicity detection of palmitic acid incubation was performed as described (35). Briefly, 100 mM palmitic acid (Sigma-Aldrich, St Louis, MO) stocks were prepared in 0.1 M NaOH at 70°C and filter sterilized. Bovine serum albumin (BSA, certified fatty acid free, low endotoxin, Sigma) was dissolved in complete media to a final concentration of 1% (w/v) and sterilized using a 0.45 μm non-pyogenic filter. Palmitic acid was added to a final concentration of 0.2, 0.4, 0.6, and 0.8 mM as different treatment levels. Control media (carrier) contained NaOH and filtered acid-free bovine serum albumin (BSA, Sigma-Aldrich).

In order to explore the impacts of palmitic acid on cell autophagy, chloroquine diphosphate salt (CQ, Sigma-Aldrich, C6628) was used in cell treatment (36, 37). GS cells were pretreated with palmitic acid for 20 h. And then, CQ was added into the culture medium at the final concentration of 5 μM and co-incubated for another 4 h.

### siRNA-Mediated FASN Knockdown

It has been reported that the biosynthesis of palmitate is catalyzed by fatty acid synthase (FAS) (38). If FAS affected SGIV replication, palmitic acid could also influence it. The FASN (GenBank Accession No.: FJ196231) siRNA was designed using Thermo Fisher online tool BLOCK-iT™ RNAi Designer (https://rnaidesigner.thermofisher.com/rnaiexpress/sort.do). GS cells were transfected with FASN siRNA (siFASN: 5′-GGGUUCAAGUCGUUGACCAGCCUAU-3′) or the same volume of negative control (NC) siRNA for 24 h, and then infected with SGIV for 24 h. Cytopathic effects (CPE) caused by SGIV infection were observed under a light microscope (Zeiss). The effects of FASN siRNA on the transcriptional levels of viral genes were evaluated by qRT-PCR.

### Cell Viability

WST-1 assay was performed to examine the impact of palmitic acid treatment on cell proliferation. In brief, cells cultured in 96-well plates were incubated with palmitic acid at indicated concentrations (0, 0.2, 0.4, 0.6, and 0.8 mM) for 24 h. Cells were washed with culture medium three times. Then, 100 μL of culture medium was filled into each well. After adding 10 μL of cell proliferation reagent WST-1 (Roche) into each well and incubation at 28°C for 4 h, the absorbance was measured in Varioskan™ LUX multimode microplate reader (Thermo Fisher, USA) at 450/655 nm.

### Virus Infection

GS cells were seeded in 24-well plates for 18 h and then incubated with palmitic acid for 24 h. After that, cells were infected with SGIV at multiplicity of infection (MOI) of 0.5. The CPE was observed under a light microscope (Zeiss). Mock- or virus-infected cells were collected for further qRT-PCR analysis, western blot, and virus titer assay.

### RNA Isolation and Real Time Quantitative PCR (qRT-PCR) Analysis

GS cells were infected with SGIV at MOI of 0.5. The total RNAs of cells were extracted using the SV Total RNA Isolation Kit (Promega) and reversed to synthesize the first-strand cDNA using the ReverTra Ace kit (Toyobo). Then the mRNA transcriptional levels of FA synthesis related genes were evaluated by qRT-PCR at 12 h post-infection (p.i.). The primers of ACC1 (Acetyl-CoA carboxylase 1), SREBP-1 (sterol regulatory element-binding protein 1), LXR (liver X receptor), and FASN gene (fatty acid synthase) are listed in Table 1.

**Table 1:** Primers used in this study.

**Name**	**Sequence (5^**′**^-3^**′**^)**
β-Actin-RT-F	TACGAGCTGCCTGACGGACA
β-Actin-RT-R	GGCTGTGATCTCCTTCTGCA
MCP-RT-F	GCACGCTTCTCTCACCTTCA
MCP-RT-R	AACGGCAACGGGAGCACTA
ICP-18-RT-F	ATCGGATCTACGTGGTTGG
ICP-18-RT-R	CCGTCGTCGGTGTCTATTC
VP19-RT-F	TCCAAGGGAGAAACTGTAAG
VP19-RT-R	GGGGTAAGCGTGAAGACT
LITAF-RT-F	GATGCTGCCGTGTGAACTG
LITAF-RT-R	GCACATCCTTGGTGGTGTTG
EcIRF3-RT-F	ATGGTTTAGATGTGGGGGTGTCGGG
EcIRF3-RT-R	GAGGCAGAAGAACAGGGAGCACGGA
EcIRF7-RT-F	CAACACCGGATACAACCAAG
EcIRF7-RT-R	GTTCTCAACTGCTACATAGGGC
EcISG15-RT-F	CCTATGACATCAAAGCTGACGAGAC
EcISG15-RT-R	GTGCTGTTGGCAGTGACGTTGTAGT
EcMDA5-RT-F	ACCTGGCTCTCAGAATTACGAACA
EcMDA5-RT-R	TCTGCTCCTGGTGGTATTCGTTC
EcMXI-RT-F	CGAAAGTACCGTGGACGAGAA
EcMXI-RT-R	TGTTTGATCTGCTCCTTGACCAT
EcIFP35-RT-F	TTCAGATGAGGAGTTCTCTCTTGTG
EcIFP35-RT-R	TCATATCGGTGCTCGTCTACTTTCA
EcTBK1-RT-F	CCTGCTGACCGACAACTGGA
EcTBK1-RT-R	GAGGCGATATTTCATGGCACA
EcTRIF-RT-F	AAACCAACCACTGGACCAAACTT
EcTRIF-RT-R	GATGGCATCCTCGACACACCTCA
EcFASN-RT-F	GGTCGGGTTCAAGTCGTT
EcFASN-RT-R	GCCTTCACTGCGTCCTCT
EcACC1-RT-F	ACTGGGGTGGTTGCTGTGG
EcACC1-RT-R	CCTTAATAGCTTGGGCTGTTTTG
EcSREBP-RT-F	TGTATCCAACTGTTGAGCACCTG
EcSREBP-RT-R	CTGTGGCAGTGTGGTCCTAG
EcLXR-RT-F	TCATGTCAGTCCAGGAGATTGTG
EcLXR-RT-R	GGTTGTACCGCCGTGATGTC

In order to determine the roles of palmitic acid in virus infection, GS cells were incubated with palmitic acid for 24 h, and infected with SGIV for 24 h at 28°C. Virus-infected cells were collected for RNA extraction and qRT-PCR analysis. The qRT-PCR analysis was performed in the QuantStudio™ 5 Real-Time PCR System (Thermo Fisher, USA). Each assay was carried out in triplicate. The mRNA expression level of viral genes, including SGIV-MCP (major capsid protein), SGIV-VP19, SGIV-ICP-18, and SGIV-LITAF (lipopolysaccharide-induced TNF-α factor) as well as the host genes, were evaluated and the primers are listed in Table 1. The data were calculated as the folds based on the expression level of targeted genes normalized to β*-*actin.

### Virus Titer Assay

Viral replication kinetics was assessed in GS cells to determine the effect of palmitic acid on SGIV production. In brief, GS cells pretreated with palmitic acid or vehicle for 24 h were infected with SGIV (at MOI of 0.5) and collected at 48 h p.i. for virus titer determination. The viral titers of cell lysates were evaluated using the 50% tissue culture infectious dose (TCID_50_) assay (39). The CPEs were observed under a light microscope (Leica, Germany) every day, and each sample was measured in triplicate.

### Western Blot Analysis

After the experimental treatments, cells were lysed and solubilized in 40 μL of Pierce IP Lysis Buffer (Thermo Fisher Scientific), containing protease/phosphatase inhibitor cocktail. Samples were boiled for 5 min after mixing with 5× loading buffer. Solubilized proteins were resolved by 6, 10, or 12% SDS-PAGE and then electrophoretically transferred to 0.2 μm Trans-Blot Turbo PVDF (Minipore). The membranes were blocked with 5% skim milk or 3% bovine serum albumin (BSA) dissolved in PBS for 2 h, then incubated with different primary antibodies overnight at 4°C. Membranes were washed for 3 times in PBST or TBST (for phosphorylation assay) buffer subsequently. Then, secondary goat-anti-rabbit or goat-anti-mouse antibody labeled with horseradish per-oxidase was used, and bound proteins were detected with Enhanced HRP-DAB chromogenic substrate Kit (TIANGEN; www.tiangen.com) according to the manufacturer's protocol. The following primary antibodies were used: anti-LC3B (1:1,000 dilution, Abcam), anti-p62 (1:1,000 dilution, Abcam), anti-Akt (1:1,000 dilution, Abcam), anti-p-Akt (Ser473) (1:1,000 dilution, Cell Signaling), anti-mTOR (1:1,000 dilution, Cell Signaling), anti-p-mTOR (1:1,000 dilution, Abcam), anti-SGIV-MCP (1:1,000 dilution), and anti-β-tubulin (1:5,000 dilution, Abcam). Data were normalized to the mean of β-tubulin expression.

### Nile Red Staining, Immune Fluorescence Assay, and Fluorescent Microscopy

Applied as a vital stain for the detection of intracellular lipid droplets, Nile Red (9-diethylamino-5H-benzo[α] phenoxazine-5-one) is characterized by red fluorescence (excitation 515–560 nm, emission >590 nm) (40, 41). The stock solution of Nile Red was prepared in acetone (0.5 mg/mL) and stored at 4°C, protected from light. After 24 h incubation with palmitic acid, GS cells were washed three times with phosphate buffer saline (PBS). After that, the cells were fixed with 4% paraformaldehyde for 30 min and then stained with Nile Red solution in the dark for 5 min at room temperature (42). The stain of intracellular lipid in GS cells was performed using a solution of Nile Red (5 μg/mL) by diluting the stock solution of dye (1:100) in PBS (41). Afterward, the cells were washed with PBS three times. The GS cells' steatosis was visualized using fluorescence microscopy (Zeiss, Germany), and the intracellular lipid vacuoles showed red fluorescence. The fluorescence intensity analysis of intracellular fat accumulation in GS cells was performed by Image J (https://imagej.en.softonic.com/). Each experiment was repeated three times.

Virus-infected GS cells or mock cells were fixed with 4% paraformaldehyde at 24 h p.i. After incubation with anti-MCP (SGIV) (1:100), cells were washed with PBS and incubated with FITC-conjugated goat anti-rabbit antibodies (Pierce). Then they were stained with Nile Red as described previously. Finally, samples were stained with 1 mg/mL 6-diamidino-2-pheny-lindole (DAPI) and observed under fluorescence microscopy (Zeiss, Germany).

### EZClick™ Palmitoylated Protein Assay

Palmitoylation is a type of post-translational modification and occurs when fatty acids like palmitic acid, are covalently attached to side chains of cysteine in proteins. Here, EZClick™ Palmitoylated Protein Assay Kit (Biovision, Catalog # K452-100) was used to detect the influence of SGIV infection on the cellular palmitic acid level. According to the protocol, Negative Control Cells are unstained cells, and cells are not exposed to Palmitic Acid Label or EZClick™ Fluorescent Azide. Background Control Cells are only exposed to EZClick™ Reaction, no EZClick™ Palmitic Acid Label. Positive Control Cells are incubated with 1× EZClick™ Palmitic Acid Label and EZClick™ Reaction. Experimental Cells are incubated with SGIV and then exposed to EZClick™ Reaction. Briefly, GS cells were seeded in 24-well plate overnight, then cells of Experimental Group were infected with SGIV (MOI = 0.5) for 12 h. For the positive control cells, media was replaced with fresh aliquots containing EZClick™ Palmitic Acid Label (1,000×) diluted to 1× final concentration with the culture medium. After 12 h incubation, 1× PBS solution was used to terminate the experiment. Then, cells were incubated with Fixative Solution for 15 min at room temperature, protected from light. After fixation, 1× PBS was used to wash the cells. Then 1× Permeabilization Buffer was added to the cells for 10 min incubation at RT. For Negative Control Cells, 400 μL of 1× PBS were added. For Background Control Cells, Positive Control Cells, and Experimental Cells, 400 μL of EZClick™ Reaction Cocktail were added, and then they were incubated for 30 min at RT protected from light. After that, the Reaction Cocktail or PBS was removed, and the cells were washed with 1× Wash Buffer three times. After staining with Hoechst33342, the cells were washed with 500 μL of ice-cold PBS and analyzed through fluorescence observation.

### Cell Transfection and Reporter Gene Assay

The effects of the palmitic acid on the promoter activity of zebrafish interferon 1 (IFN1) and interferon sequence response element (ISRE) were evaluated by reporter gene assays (43). In brief, GS cells were cultured in 24-well plates before co-transfecting with 0.8 mg ISRE-Luc/IFN1-Luc and 0.05 mg pRL-SV40 Renilla luciferase vector. At 24 h post-transfection, cells were incubated with palmitic acid, then collected at 48 h post-transfection. Luciferase activity in total cell lysates was measured by luciferase reporter assay (Promega, USA) using a Varioskan™ LUX multimode microplate reader (Thermo Fisher, USA). Co-transfection assay was carried out as described previously by using pcDNA-flag-EcIRF3/EcIRF7/EcTBK1/EcMDA5.

### Statistical Analysis

Results were expressed as means±SD. Statistical comparisons were conducted using the Student's *t*-test, and a *p*-value of < 0.05 was considered to be statistically significant.

## Results

### Palmitic Acid Involved in Fish Viral Infection

**Figure 1:**
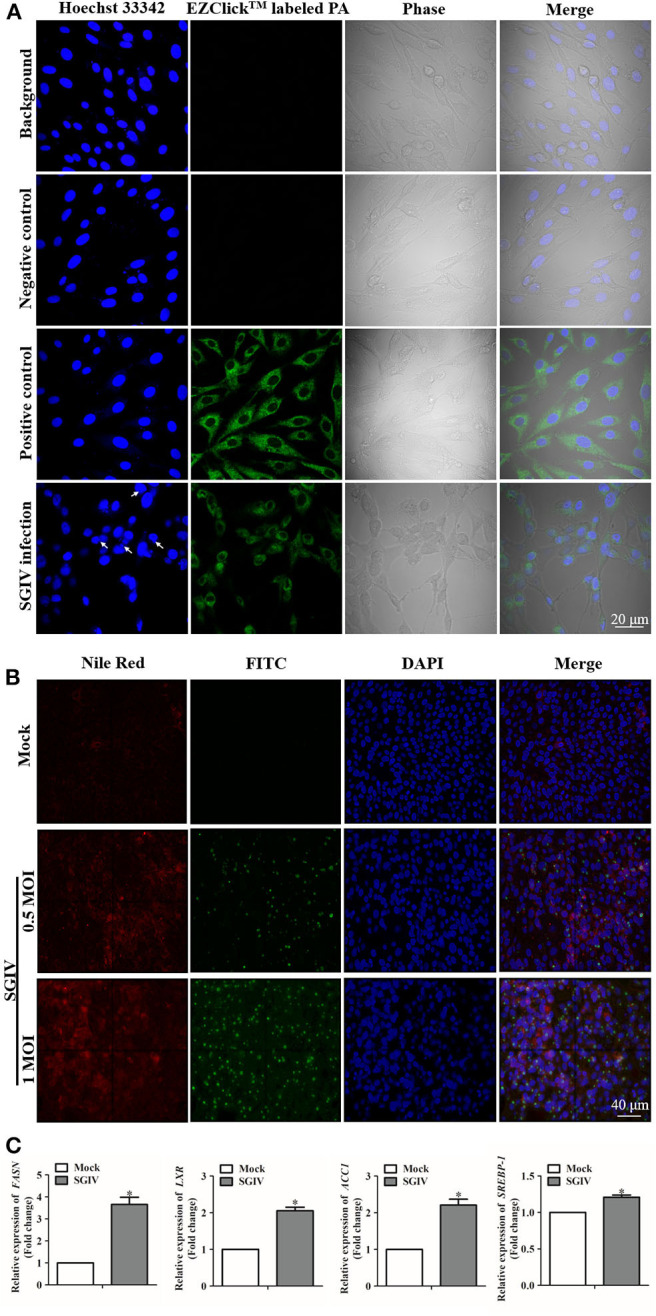
Lipids were involved in fish virus infection. **(A)** Intracellular palmitic acid accumulation analysis using EZClick™ Palmitoylated Protein Assay. The green fluorescence generated by EZClick™ Palmitic Acid was observed under the fluorescence microscope (Zeiss), and viral assembly sites are indicated by arrows. **(B)** Nile red staining of the intracellular lipids with and without SGIV infection and immunofluorescence assay carried out at the same time with the anti-SGIV-MCP antibody. The red fluorescence represents the accumulation of lipids, and green fluorescence is for SGIV-MCP. Fluorescence signals were observed under the fluorescence microscope (Zeiss). **(C)** Evaluation of the mRNA transcription levels of fatty acid synthesis related genes after SGIV infection. GS cells were infected with SGIV (MOI: 0.5), the mRNA transcriptional levels of fatty acid synthesis related genes were evaluated by qRT-PCR at 12 h post-infection. The data are represented as mean ± SD, and the statistical significances were determined with Student's *t*-test, *n* = 3. The significance level was defined as *p < 0.05.

To demonstrate whether fatty acids were associated with SGIV infection in GS cells, we first detected the changes of fatty acid synthesis after SGIV infection. The evaluation of palmitoylated protein staining suggested the lipid synthesis was promoted by SGIV infection in GS cells (Figure 1A). Virus infection increased the accumulation of lipid products, especially palmitic acid, in GS cells. As shown in Figure 1A, there was strong green fluorescent signal in the cytoplasm of positive control cells, but no signal in negative control ones. In SGIV infected cells, green fluorescence was observed in cytoplasm, which suggested that palmitoylated protein was accumulated (Figure 1A). Figure 1B shows that besides palmitic acid, SGIV infection could increase the production of other intracellular lipids. Our previous study on lipid metabolic profile of SGIV infected cells also suggested SGIV infection stimulated the accumulation of palmitic acid and at least two other fatty acids, oleic acid and eicosatetraenoic acid (unpublished data). Thus, we speculated that SGIV could utilize the lipid synthesis systems and control the cellular palmitoylation for its replication.

**Figure 2:**
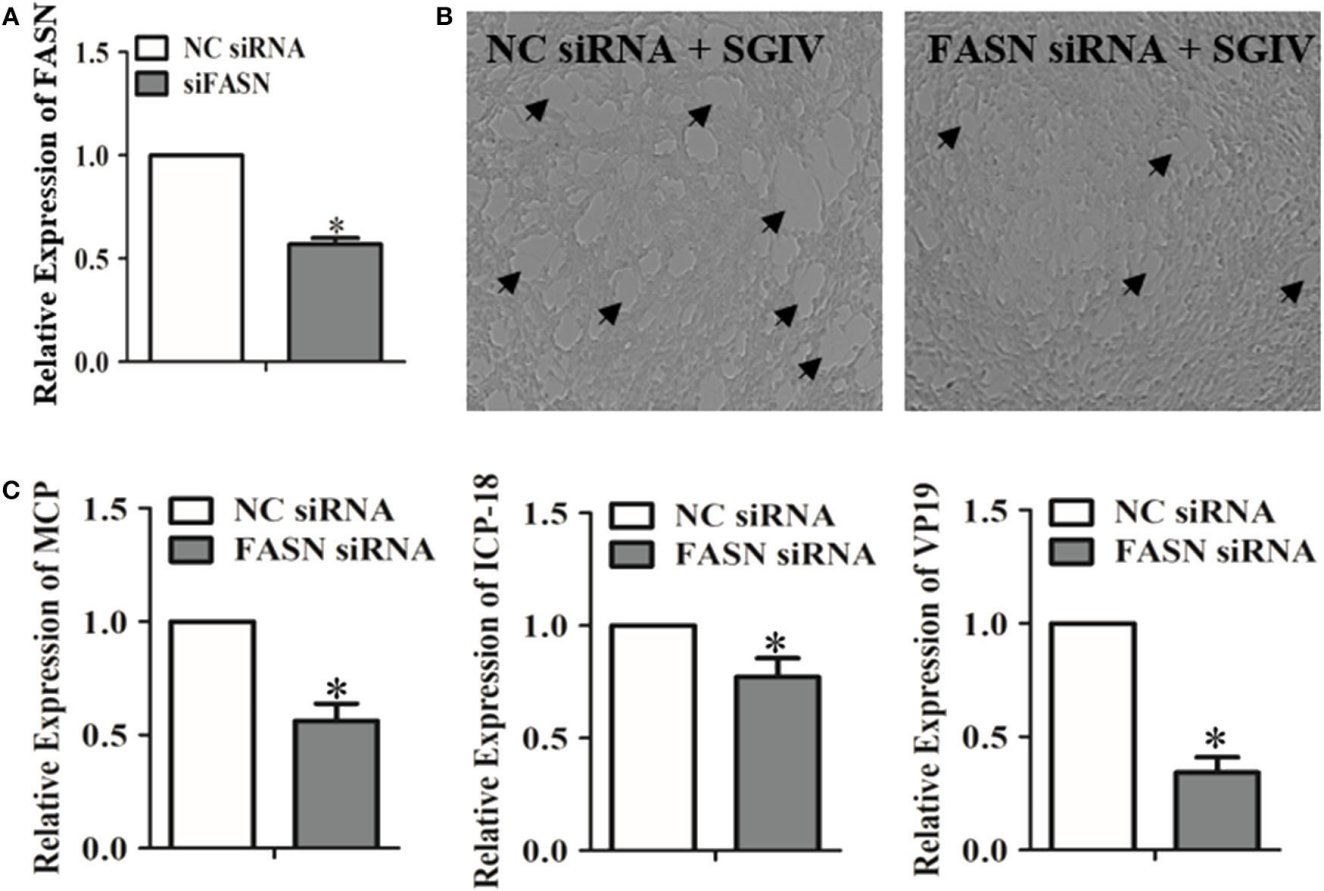
Evaluation of the impacts of the FASN gene knockdown on SGIV replication. **(A)** The interfering efficiency of the FASN gene in GS cells was analyzed by qRT-PCR. **(B)** GS cells were transfected with negative control (NC) siRNA or FASN siRNA. Cells were infected with SGIV subsequently. The cytopathic effects of SGIV infection were observed. **(C)** The mRNA transcriptional levels of SGIV functional genes were evaluated in NC siRNA or FASN siRNA transfected cells. The data are represented as mean ± SD, and the statistical significances were determined with Student's *t*-test, *n* = 3. The significance level was defined as *p < 0.05.

Genes correlated with lipids played essential roles in regulating the metabolism of lipids and participated in virus infection (44). The up-regulated levels of fatty acid regulating genes due to SGIV infection indicated that these genes could have a positive effect on SGIV infection (Figure 1C). Functioning as the catalyst in palmitate biosynthesis processes (38, 45, 46), FAS was also induced by SGIV in GS cells. Then, RNA interfering assay was carried out to knock down the fatty acid synthase gene (FASN) (Figure 2A), leading to inhibition in virus replication of SGIV (Figures 2B,C). The CPE of SGIV infection was less severe in FASN knock down cells (Figure 2B). Similarly, the mRNA level of SGIV functional genes was reduced in FASN siRNA transfection group (Figure 2C). These results suggested FASN gene was essential for SGIV replication. An earlier report had revealed that fatty acid synthase (FAS) encoded by FASN gene was a multi-functional enzyme that catalyzed palmitate biosynthesis in a NADPH-dependent reaction (38). However, the mechanisms of palmitate in modulating SGIV infection and replication remain unclear. Functional analysis should be carried out to investigate the role of palmitic acid in host-virus interaction.

### Determination of the Suitable Concentration for Cell Incubation

**Figure 3:**
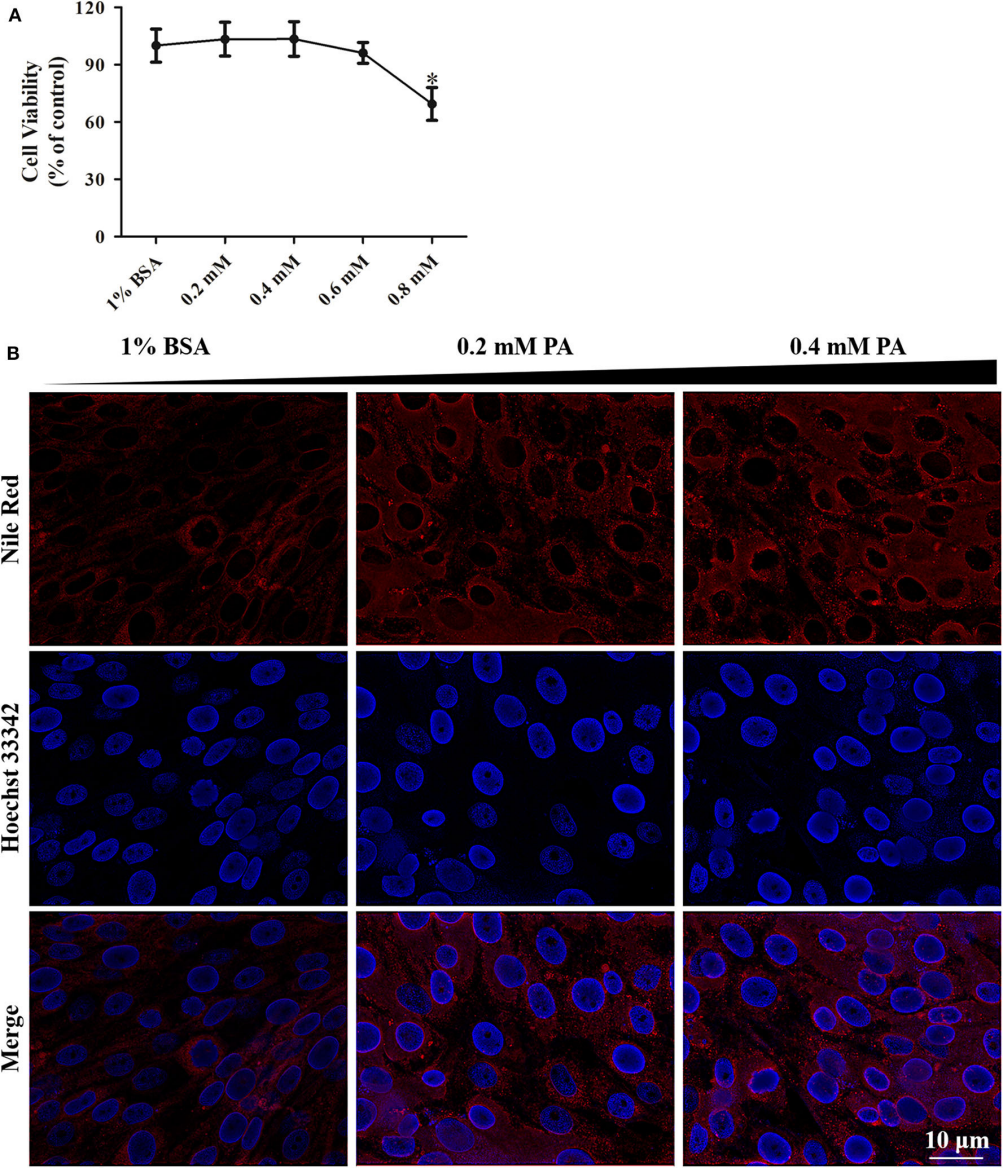
Palmitic acid treatment had low cytotoxicity in GS cells. **(A)** WST-1 assay suggesting the effect of intracellular fat accumulation (dose-dependent) on cellular cytotoxicity of GS cells in culture. Cell viability is expressed as % of control cells (1% BSA). **(B)** Fluorescence microscopy showing the accumulation of lipids intracellular at 24 h post-treatment by Nile Red staining (red). Cell nuclear was stained (blue) using Hoechst33342. The images were taken at 40× magnifications.

Changes of metabolic profile of GS cells in response to SGIV infection were documented. Palmitic acid massively increased in SGIV infected cells (unpublished data). To investigate the potential functions of palmitic acid in SGIV infection, we utilized an *in vitro* model in which GS cells were loaded with palmitic acid as previously described (3, 35). Palmitic acid showed no adverse effect on cell viability when the concentration was lower than 0.6 mM within 24 h incubation (Figure 3A). But 0.6 mM palmitic acid was toxic when incubating for 72 h or more (data no shown). A microscopic evaluation of GS cells after incubation with palmitic acid showed that the cytoplasm of palmitic acid pretreated cells contained numerous different-sized fluorescent bodies (stained with Nile Red) corresponding to lipid accumulation, i.e., steatosis (Figure 3B). In the cytoplasm of control cells (1% BSA), the presence of moderate micro- and macro-vacuolar steatosis was also observed (Figure 3B).

### Palmitic Acid Enhanced SGIV Replication

**Figure 4:**
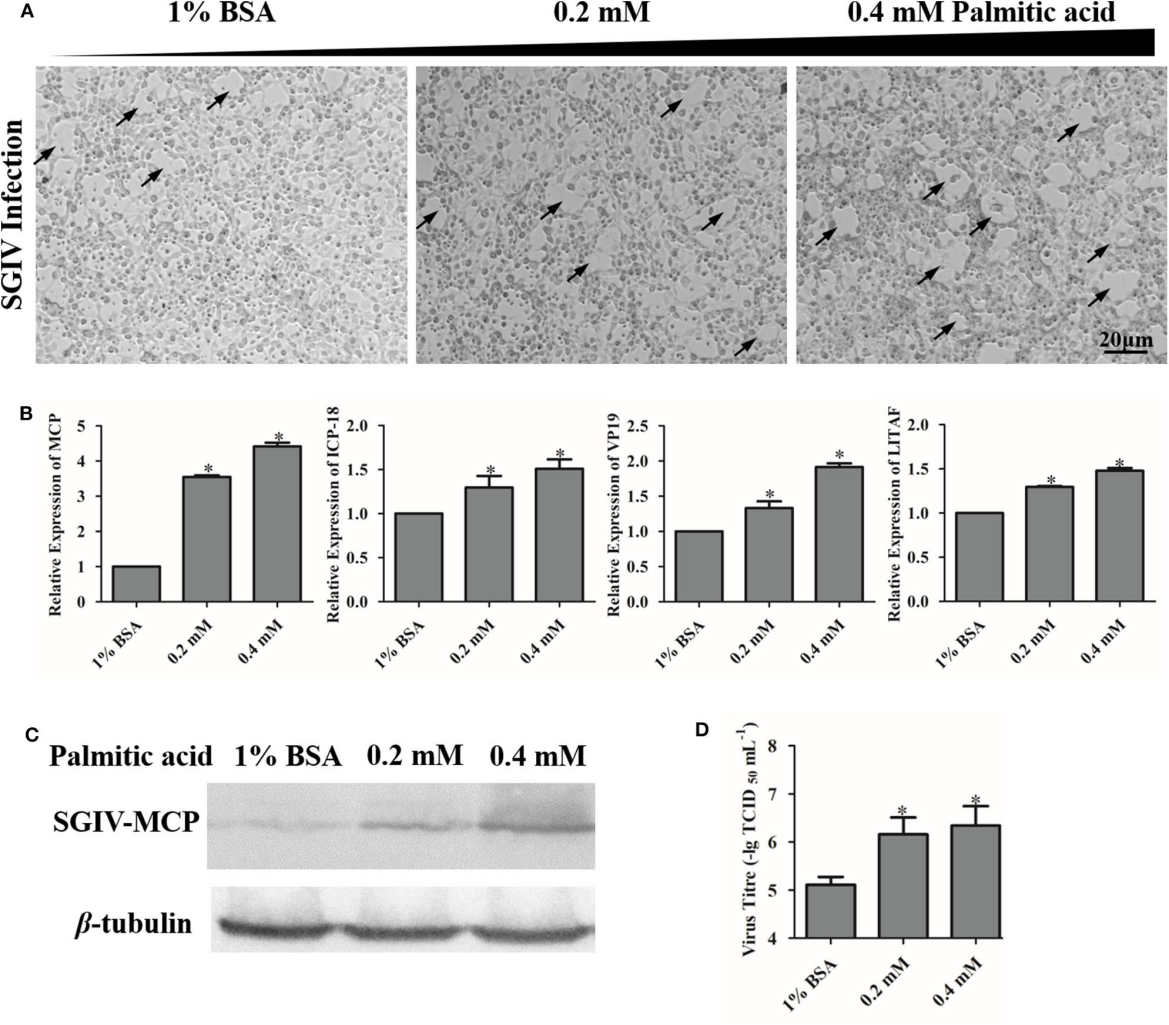
Palmitic acid enhanced SGIV replication. **(A)** The severity of CPE induced by SGIV infection in palmitic acid incubated cells was observed under the microscope (Zeiss). The arrows show the CPE induced by SGIV infection. **(B)** The relative expressions of SGIV-MCP, SGIV-LITAF, SGIV-ICP-18, and SGIV-VP19 genes after SGIV infection were evaluated by qRT-PCR. The relative expression ratio of the selected gene vs. β*-*actin (reference gene) was calculated using the 2^−ΔΔCT^ method. **(C)** Virus protein level increased after palmitic acid treatment. The level of SGIV-MCP was detected by western blot, and β-tubulin was used as the internal control. **(D)** Virus production of SGIV was evaluated. GS cells incubated with indicated concentration palmitic acid (0, 0.2, or 0.4 mM palmitic acid) were infected with SGIV and collected at 24 h p.i. Viral titers were determined using the TCID_50_ method. The data are represented as mean ± SD, and the statistical significances were determined with Student's *t*-test, *n* = 3. The significance level was defined as *p < 0.05.

To investigate the effects of palmitic acid on SGIV virus infection, we evaluated the CPE progression and detected the viral gene transcription as well as the viral coat protein synthesis of SGIV in infected palmitic acid loading cells. As shown in Figure 4, SGIV infection induced CPE was more severe in palmitic acid treated cell groups than in untreated cells (Figure 4A). Moreover, in palmitic acid loaded cells, the transcription level of SGIV-MCP, SGIV-ICP-18, SGIV-VP19, and SGIV-LITAF was significantly increased (Figure 4B). And the synthesis of SGIV MCP detected by western-blotting also showed that palmitic acid improved the expression of SGIV major coat protein (Figure 4C). Virus titer assay showed increased viral replication of SGIV after palmitic acid treatment (Figure 4D). The results of virus titer assay also suggested that exogenous addition of palmitic acid enhanced SGIV replication (Figure 4D).

### Palmitic Acid Suppresses Autophagic Flux in GS Cells

**Figure 5:**
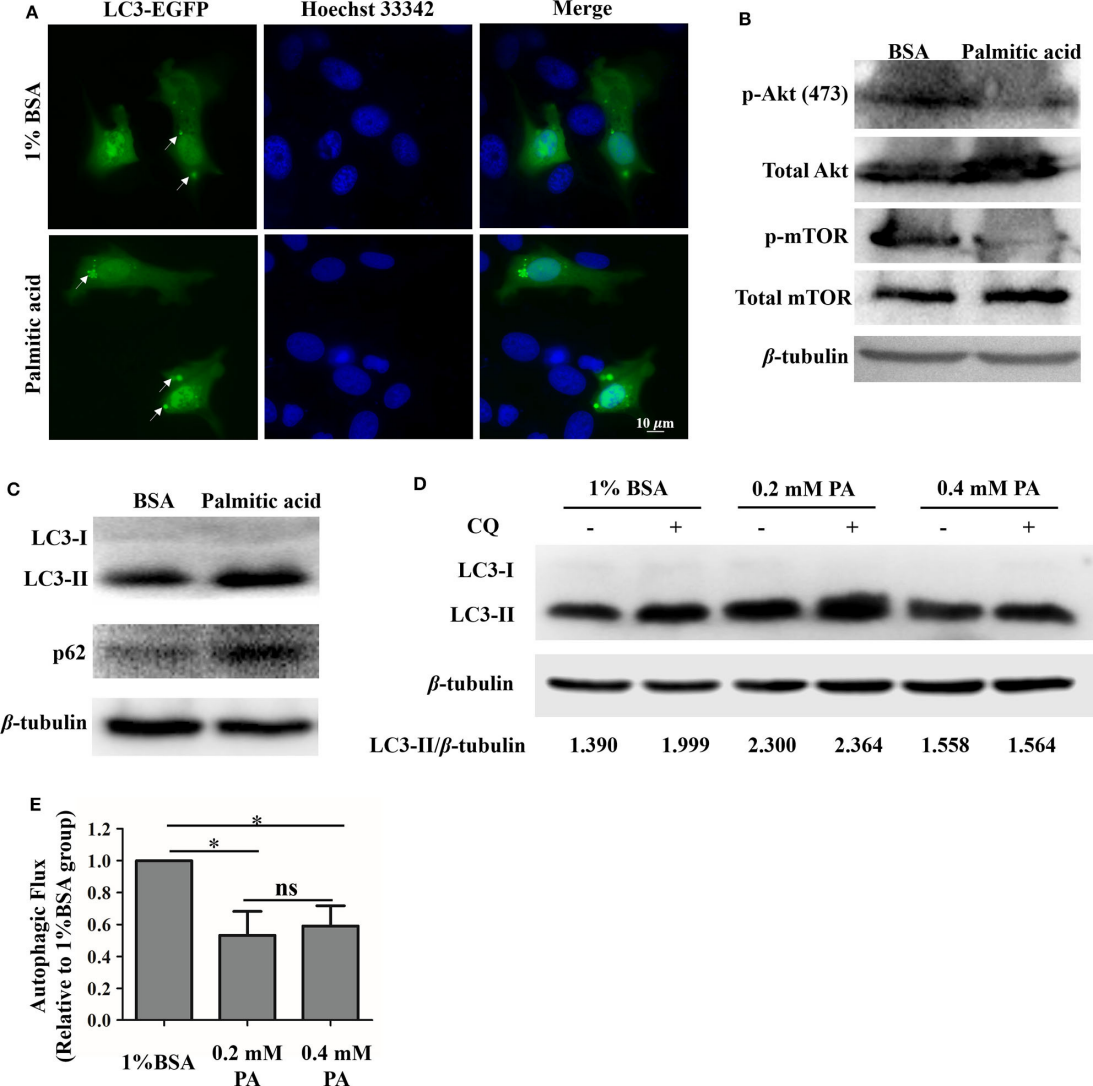
Palmitic acid impacted autophagic flux in GS cells. **(A)** Accumulation of GFP-LC3 (green) were observed after palmitic acid treatment. GS cells were transfected with C1-EGFP-LC3 plasmid, then incubated with or without 0.4 mM palmitic acid. Cells were fixed with 4% polyformaldehyde and staining with Hoechst33342. Fluorescence was observed under the fluorescence microscope (Zeiss). **(B)** Representative image of phospho-Akt (Ser473) [p-Akt (ser473)], and phospho-mTOR (p-mTOR) detection was performed to verify Akt and mTOR inhibition by 1% BSA or palmitic acid (0.4 mM) treatment. β-tubulin was used as the internal control. **(C)** The expression levels of LC3, and p62 in cell lysates were evaluated after the incubation of 1% BSA or palmitic acid (0.4 mM) for 24 h. Western blot assay was carried out, and β-tubulin was used as the internal control. **(D)** GS cells were incubated with 1% BSA, 0.2 mM palmitic acid, or 0.4 mM palmitic acid for 24 h, and CQ was added for the last 4 h treatment. Western blot assay was carried out to detect the LC3-II and β-tubulin levels in cell lysate. Band intensity was calculated using Image J software, and the LC3-II protein level was presented by the ratio of LC3-II/β-tubulin. **(E)** Autophagic flux was measured. Briefly, after measuring the LC3-II protein level (LC3-II/β-tubulin) in each group, the histogram was made referring to the ratio of LC3-II level in cells treated with CQ to that of untreated cells. Setting the ratio in 1% BSA treated group as 1-fold. The data are represented as mean ± SD, and the statistical significances were determined with Student's *t*-test, *n* = 3. The significance level was defined as *p < 0.05.

It has been demonstrated that palmitic acid can modulate autophagy in many kinds of cells. We sought to determine whether palmitic acid could also modify this process in GS cell (5, 47). Firstly, the green fluorescence of LC3 was observed under a fluorescence microscope with and without palmitic acid treatment. As shown in Figure 5, increased levels of green signal of LC3, as well as the higher levels of LC3-II and p62 in the palmitic acid treated group evidenced by western blot assay indicate that palmitic acid induces the accumulation of autophagosomes in GS cells (Figures 5A,C). Autophagy is known to be a dynamic process, so the detection of LC3-II levels is not sufficient to assess autophagic activity in cells (5, 48). The inhibition of palmitic acid on p-Akt (Ser473) and p-mTOR was evaluated (Figure 5B). It has been highlighted that the reduced level of p-Akt and p-mTOR could contribute to the promotion of autophagy (49). However, the increased level of LC3-II may be due to the increased autophagic activity, or the blockades of lysosomal function (5, 48). To discriminate between these two possibilities, we determined the impacts of palmitic acid on autophagic flux using chloroquine (CQ), an inhibitor of autophagosome-lysosome fusion. GS cells were incubated with vehicle (1% BSA), 0.2 mM palmitic acid, or 0.4 mM palmitic acid for 24 h, and CQ was added for the last 4 h treatment. The pixel values of LC3-II and β-tubulin were evaluated using Image J. Then, the autophagic flux was measured as the ratio of LC3-II level (LC3-II/β-tubulin) in cells treated with CQ to that of untreated cells as described previously (5). As shown in Figure 5E, the calculated result was performed in histogram. The autophagic flux was blocked in the presence of palmitic acid (Figure 5E). Taken together the decreased autophagic flux and the accumulation of LC3-II and p62 caused by palmitic acid, we subsequently concluded that palmitic acid decreased autophagy flux by inhibition of autophagosome–lysosome fusion step.

### Palmitic Acid Negatively Regulated the Interferon Signaling Molecules

**Figure 6:**
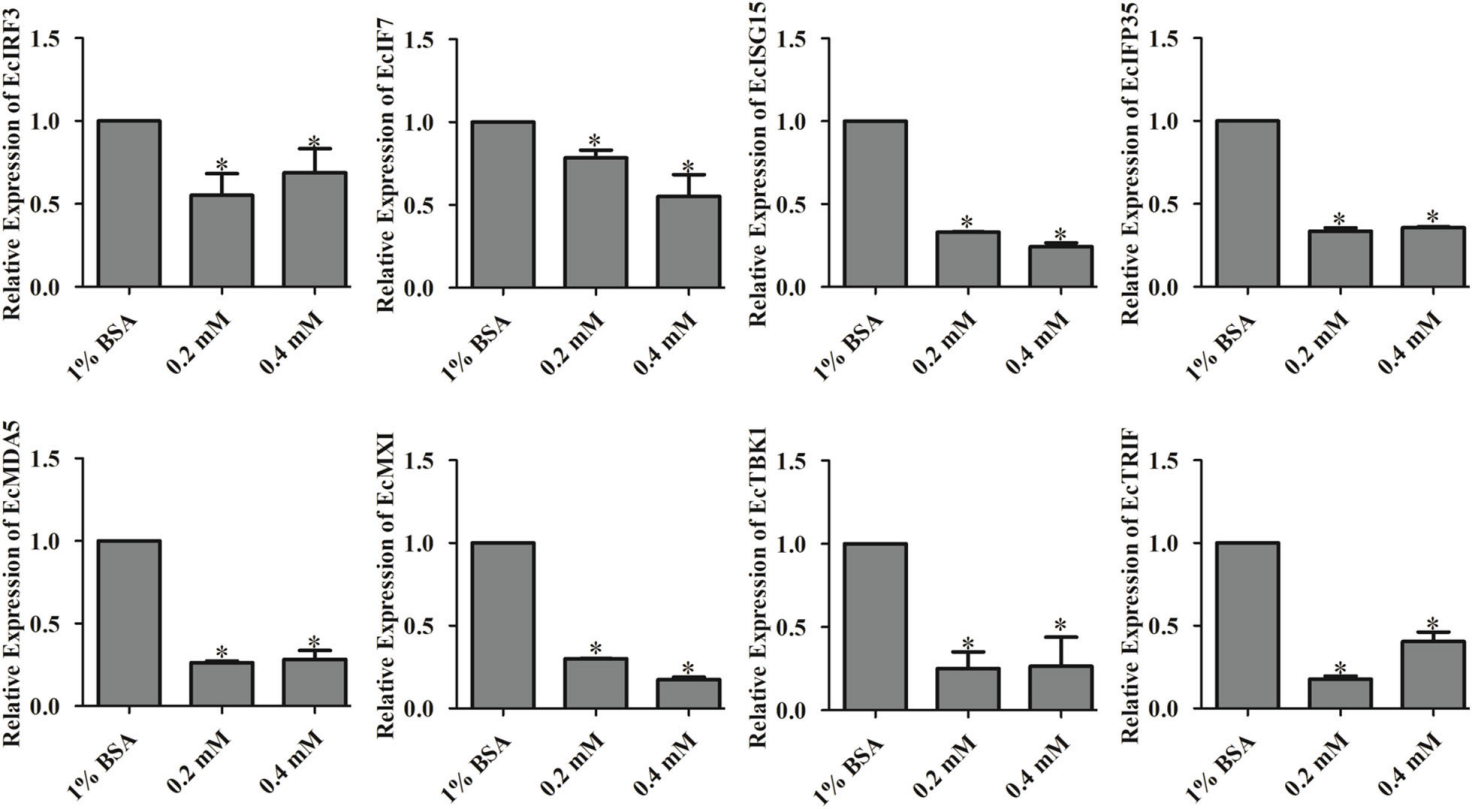
Palmitic acid decreased the expression of interferon related cytokines or effectors. GS cells were incubated with the indicated concentration of palmitic acid (0.2 or 0.4 mM) or 1% BSA, then harvested at 24 h post-incubation. Expression levels of host IFN associated genes were determined using qRT-PCR. The data are represented as mean ± SD, and the statistical significances were determined with Student's *t*-test, *n* = 3. The significance level was defined as *p < 0.05.

To determine the regulatory effects of palmitic acid on the expression of interferon related molecules, we detected the transcripts of these genes in GS cells treated with different concentrations of palmitic acid. As shown in Figure 6, the relative expression of EcIRF3 (interferon regulatory factors 3), EcIRF7, EcISG15 (interferon-stimulated gene), EcIFP35 (Interferon-induced 35 kDa protein), EcMXI (Interferon-induced GTP-binding protein Mx1), EcTBK1 (TANK-binding kinase 1), EcTRIF (TIR-domain-containing adapter-inducing interferon-β), and EcMDA5 (melanoma differentiation-associated protein 5) were significantly decreased in palmitic acid treated cells compared to the control cells, suggesting that palmitic acid inhibited the interferon responses (Figure 6).

**Figure 7:**
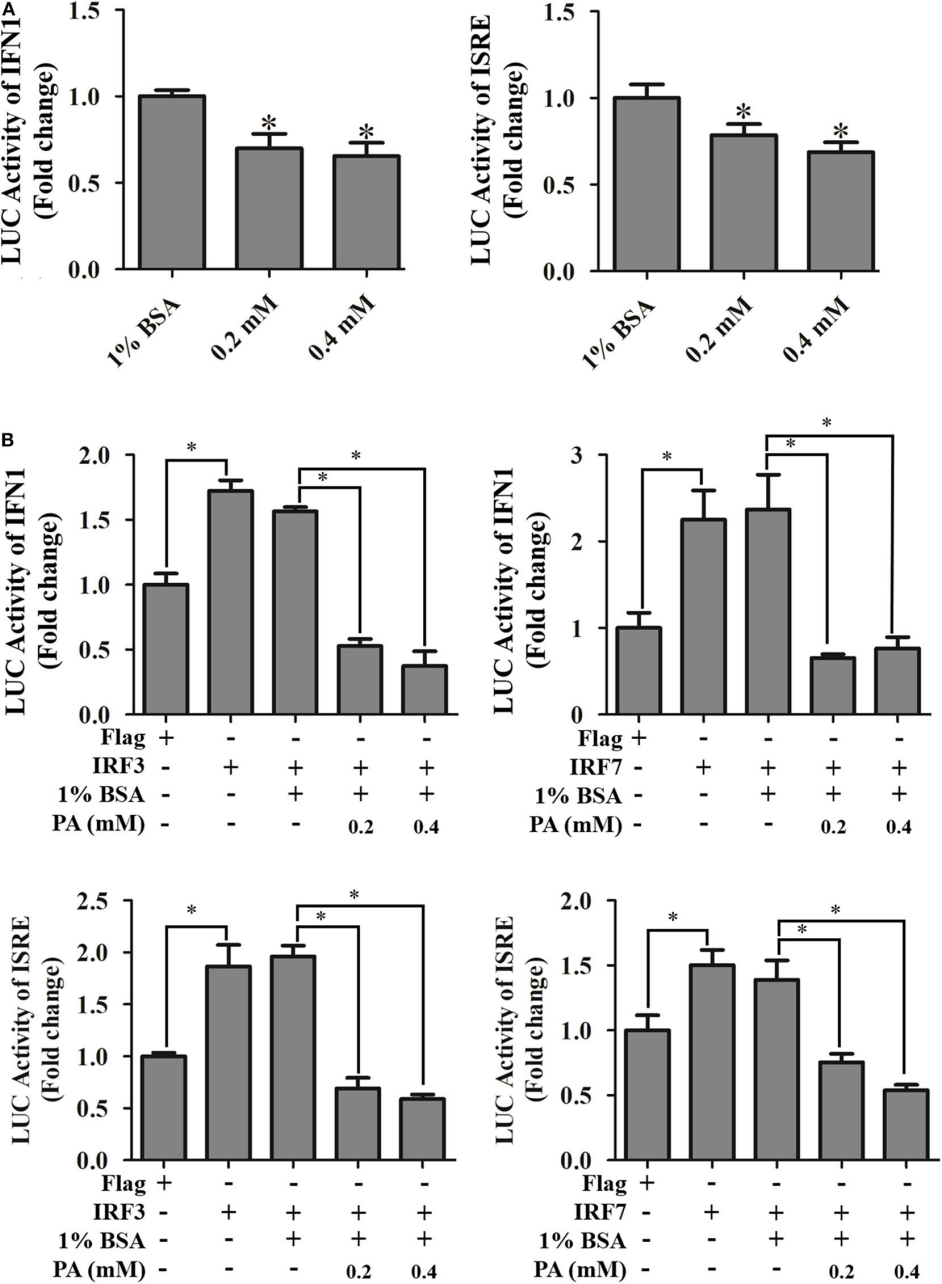
Effects of palmitic acid on IFN1 promoter, and ISRE promoter activity. **(A)** Treatment of palmitic acid decreased promoter activities of IFN1 and ISRE. GS cells were co-transfected with IFN1-Luc/ISRE-Luc and pRL-SV40 Renilla luciferase vector, and treated with 1% BSA or indicated concentration of palmitic acid (0.2 or 0.4 mM), respectively. The promoter activity was measured using the luciferase reporter gene assay. Setting promoter activity in 1% BSA treated group as 1-fold. **(B)** Palmitic acid reduced IRF3/7 evoked IFN1 and ISRE activity. After transfection, GS cells were incubated with 1% BSA or 0.2 mM, 0.4 mM palmitic acid. Luciferase vs. Renilla luciferase activities in cell lysates was measured and expressed as the fold stimulation. Setting promoter activity in Flag transfected group as 1-fold. All data are representative of three independent experiments. The data are represented as mean ± SD, and the statistical significances were determined with Student's *t*-test, *n* = 3. The significance level was defined as *p < 0.05.

On the other hand, the effects of palmitic acid treatment on IFN and ISRE promoter activities were evaluated using reporter gene assay. Palmitic acid treatment not only decreased the IFN and ISRE promoter activities (Figure 7A) but also suppressed IRF3/IRF7 evoking interferon signaling pathway (Figure 7B).

**Figure 8:**
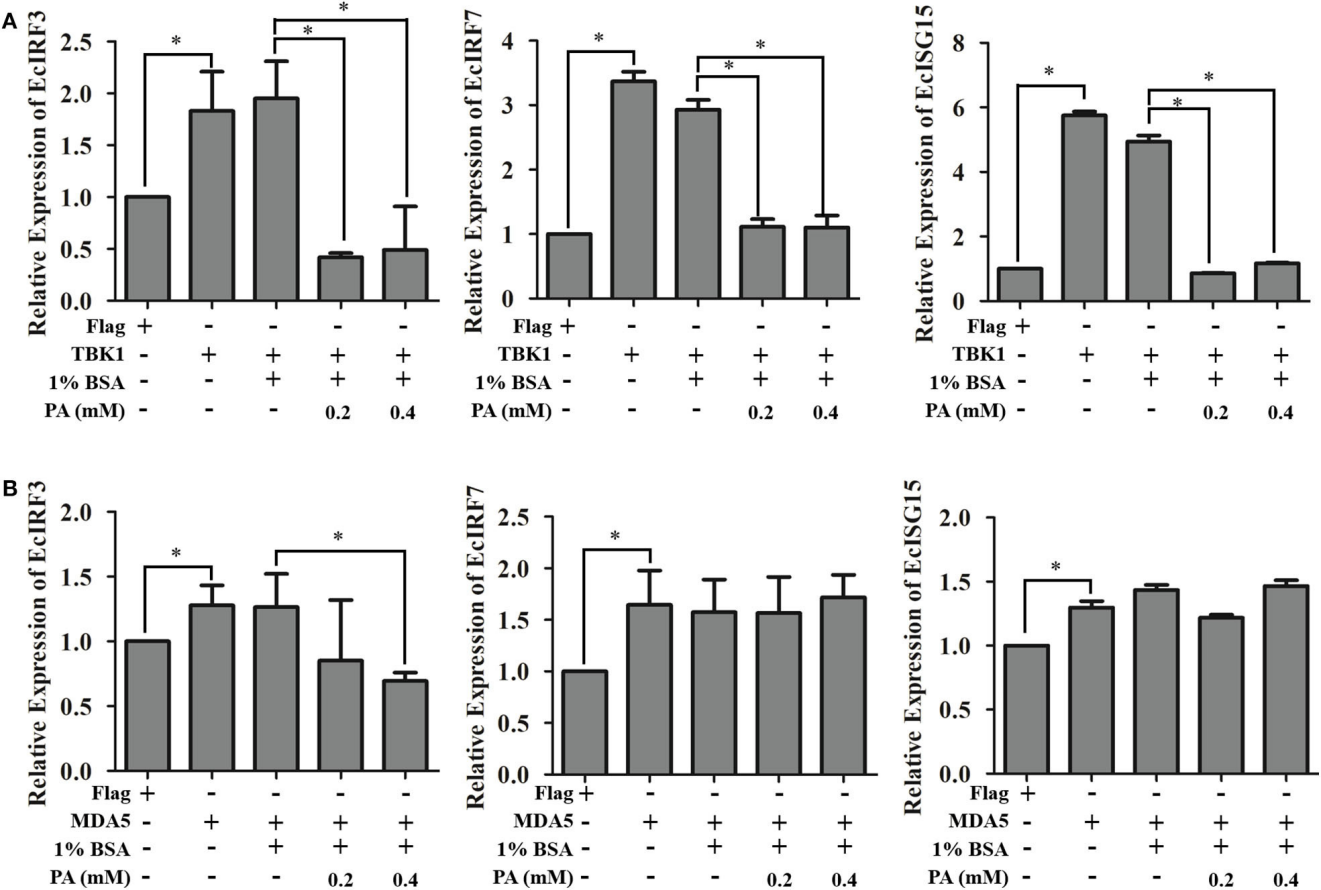
Palmitic acid decreased TBK1-, but not MDA5-induced interferon immune response. GS cells were transfected with EcTBK1 **(A)** or EcMDA5 **(B)** and then incubated with 1% BSA or palmitic acid at indicated concentrations (0.2 or 0.4 mM). The transcription of interferon related genes, including EcIRF3, EcIRF7, and EcISG15, were detected using qRT-PCR. Setting the mRNA expression level in Flag transfected cells as 1-fold. All data are representative of three independent experiments. The data are represented as mean ± SD, and the statistical significances were determined with Student's *t*-test, *n* = 3. The significance level was defined as *p < 0.05.

Furthermore, the insights of palmitic acid modulations were discovered by detecting its regulation of MDA5-/TBK1-induced interferon signaling pathway. Transcriptional levels of EcIRF3, EcIRF7, and EcISG15 were detected using qRT-PCR method. As shown in Figure 8, palmitic acid significantly decreased the *TBK1*-induced interferon responses. But there were no significant differences between BSA or palmitic acid incubated groups under MDA5 transfection. Thus, we proposed that palmitic acid treatment could down-regulate IFN signaling by inhibiting the TBK1-IRF3/7 pathway.

## Discussion

As structural elements of viral and cellular membranes, lipids were suggested to be involved in the intricate virus-cell interaction in many ways (50). Results obtained with a variety of viruses suggested that lipids played significant roles in cell metabolism, fatty acid synthesis and generation of a specific lipid microenvironment enriched in phosphatidylinositol 4-phosphate (PI4P) for efficient viral replication (10, 51, 52). Identifying the influences of bioactive lipid mediators on host inflammation, viral replication, and disease progression would lead to the discovery of lipid-active compounds as potential antiviral drugs and the development of antiviral strategies (53). Numerous studies have been cited to illustrate that viral infection was associated with lipid metabolomics (54, 55). Palmitic acid, the product of the FAS-mediated biosynthesis process, was up-regulated during SGIV infection indicated by the lipid metabolism profiles (unpublished data). In the current study, we have demonstrated that SGIV infection triggered the synthesis and accumulation of intracellular palmitic acid as well as the fatty acid regulating genes. Additionally, knockdown of the FASN gene suppressed the infection and replication of SGIV *in vitro*. FASN gene can be induced by white spot syndrome virus (WSSV) infection in shrimps, and facilitate virion formation and viral morphogenesis (56). Moreover, our previous study reveals FASN gene is critical for RGNNV replication (57). In the current study, the suppression of SGIV replication caused by FASN knockdown also suggested the essential role of FASN gene for SGIV pathogenesis. Hence, we speculated that the up-regulated FASN expression level by SGIV would induce palmitic acid, and subsequently contribute to the progress of SGIV infection in fish cells.

As a saturated fatty acid, palmitic acid plays dual roles in promoting cell growth and inducing cell death (58). For instance, palmitic acid promotes astrocytogenesis in the differentiated neural stem cells (59), and also stimulated hepatocyte proliferation (60). But the high concentration of palmitic acid (1 mM) significantly decreased HepG2 cell viability (61). In our study, no adverse effects toward GS cells were detected when incubated with palmitic acid under the concentration of 0.6 mM within 48 h incubation. Evidenced by the severity of CPE, increased levels of viral genes, and viral proteins expression, as well as higher viral titer levels, palmitic acid was confirmed to promote SGIV infection and replication. It had been highlighted that palmitic acid and oleic acid co-treatment led to defective Jak-Stat signaling and blocked the antiviral activity of interferon-alpha against the hepatitis C virus (HCV) *in vitro* (62). Accordingly, the reduction of mRNA transcriptional level of interferon related signaling molecules, including EcIRF3, EcIRF7, EcISG15, EcIFP35, and EcMXI, implied palmitic acid exerted negative regulation on interferon antiviral immune in fish cells. Further analysis showed that palmitic acid significantly inhibited TANK-binding kinase-1 (TBK1), but not MDA5-inducing interferon response. EcTBK1 was identified as a vital inhibitor in the viral infection processes of SGIV, by triggering the IRF3- and IRF7-regulated interferon promotor ISRE and IFN activity in our previous research (63). Thus, palmitic acid might restrict the interferon response through its influence on the TBK1-IRF3/7 signaling pathway in fish.

Autophagy is an essential mechanism in cell survival under certain stress conditions, such as nutrient deprivation (64). However, under some circumstances, uncontrolled massive autophagy promoted cell death and has been described as type II programmed cell death (PCD) (65). A variety of DNA and RNA viruses induce PCD actively during infection, which is critical in the pathogenesis of viral diseases (66, 67). Evidence also revealed that autophagy induced by Zika virus (ZIKV) through its inhibition of Akt-mTOR signaling in human fetal neural stem cells, resulted in the increased virus replication level and impeded neurogenesis (68). In the hepatitis B virus (HBV) infection, glucosamine can act as the promotor of virus replication by inducing autophagic stress through its double effects in suppressing autophagic degradation and inhibiting mTORC1 signaling pathway (69). Reports suggest that palmitic acid impacts on cell autophagy, the steward of cellular process (4, 5).

In the current study, accumulation of intracellular LC3 was observed, and the reduction of the p-Akt (Ser473) and p-mTOR phosphorylation also suggested palmitic acid is associated with cell autophagy (49). Further study revealed the increased LC3-II and p62 proteins level after palmitic acid treatment. As a substrate of autophagy-mediated degradation, p62 protein plays roles in cell survival, cell death, cell proliferation, and tumorigenesis (70–72). Impairments in autophagy are usually accompanied by a massive accumulation of p62 protein (73). Additionally, known as the phosphatidylethanolamine conjugate of LC3-I, LC3-II is recruited to autophagosomal membranes where it binds to p62 (74). The increased protein level of LC3-II suggests an increased formation or accumulation of autophagosomes (5). In order to discriminate the effect of palmitic acid between these two possibilities, GS cells were treated with CQ as described previously (74, 75). The ratio between LC3-II protein level in the presence and absence of CQ, which indicates autophagic flux, was significantly reduced by palmitic acid (5, 75). Together, our results suggested the blockade of autophagic flux by palmitic acid through suppressing the fusion of autophagosome-lysosome.

Lipid interactions, including membrane envelopment, membrane fusion, membrane remodeling, and signaling molecule functions, were proved to be crucial for viral replication (13, 14, 50). Increasing research on lipids in aquaculture had been illuminating. 2-HOM has been characterized as an inhibitor of SGIV that benefits from its interfering with SGIV-ORF088L myristoylation, and resistance against SGIV entry and replication (76). Exploiting specific lipid requirements of pathogens, and delineating the intricate interactions of these pathogens with cellular lipids and the modification of their metabolism, might provide new approaches for antiviral therapies (50). Here, we present a study of palmitic acid, functioning as a suppressor of autophagic flux, as well as a signaling mediator to promote viral infection and replication by inhibiting interferon signaling molecules, and down-regulating the TBK1-IRF3/7 signaling pathway. Our findings provide new mechanistic insights linking lipids and immunity in virus infections.

## Data Availability Statement

The raw data supporting the conclusions of this article will be made available by the authors, without undue reservation.

## Author Contributions

YY carried out the main experiments, analyzed the data, and drafted the manuscript. CL and FZ participated in the qRT-PCR experiments and western blotting assay. JL participated in the immunofluorescence experiment. YY, QQ, XH, and YH designed the experiments and reviewed the manuscript. All authors read and approved the final manuscript.

## Conflict of Interest

The authors declare that the research was conducted in the absence of any commercial or financial relationships that could be construed as a potential conflict of interest. The reviewer JC declared a past co-authorship with several of the authors YY, SW, YH, XH, and QQ to the handling Editor.

## References

[1] Chawla A Repa JJ Evans RM Mangelsdorf DJ. Nuclear receptors and lipid physiology: opening the X-files. Science. (2001) 294:1866–70. 10.1126/science.294.5548.1866 11729302

[2] Clarke SD. The multi-dimensional regulation of gene expression by fatty acids: polyunsaturated fats as nutrient sensors. Curr Opin Lipidol. (2004) 15:13–8. 10.1097/00041433-200402000-00004 15166803

[3] Ricchi M Odoardi MR Carulli L Anzivino C Ballestri S Pinetti A. Differential effect of oleic and palmitic acid on lipid accumulation and apoptosis in cultured hepatocytes. J Gastroenterol Hepatol. (2009) 24:830–40. 10.1111/j.1440-1746.2008.05733.x 19207680

[4] Hernández-Cáceres MP Toledo-Valenzuela L Díaz-Castro F Ávalos Y Burgos P Narro C. Palmitic acid reduces the autophagic flux and insulin sensitivity through the activation of the Free Fatty Acid Receptor 1 (FFAR1) in the hypothalamic neuronal cell line N43/5. Front Endocrinol (Lausanne). (2019) 10:176. 10.3389/fendo.2019.00176 30972025 PMC6446982

[5] Ortiz-Rodriguez A Acaz-Fonseca E Boya P Arevalo MA Garcia-Segura LM. Lipotoxic effects of palmitic acid on astrocytes are associated with autophagy impairment. Mol Neurobiol. (2019) 56:1665–80. 10.1007/s12035-018-1183-9 29916142

[6] Klionsky DJ. Autophagy: from phenomenology to molecular understanding in less than a decade. Nat Rev Mol Cell Biol. (2007) 8:931–7. 10.1038/nrm2245 17712358

[7] Hirata Y Ikeda K Sudoh M Tokunaga Y Suzuki A Weng L Self-enhancement of hepatitis C virus replication by promotion of specific sphingolipid biosynthesis. PLoS Pathog. (2012) 8:e1002860 10.1371/journal.ppat.1002860 22916015 PMC3420934

[8] Li Q Pène V Krishnamurthy S Cha H Liang TJ. Hepatitis C virus infection activates an innate pathway involving IKK-alpha in lipogenesis and viral assembly. Nat. Med. (2013) 19:722–9. 10.1038/nm.3190 23708292 PMC3676727

[9] Munger J Bennett BD Parikh A Feng XJ McArdle J Rabitz HA. Systems-level metabolic flux profiling identifies fatty acid synthesis as a target for antiviral therapy. Nat Biotechnol. (2008) 26:1179–86. 10.1038/nbt.1500 18820684 PMC2825756

[10] den Boon JA Ahlquist P. Organelle-like membrane compartmentalization of positive-strand RNA virus replication factories. Annu Rev Microbiol. (2010) 64:241–56. 10.1146/annurev.micro.112408.134012 20825348

[11] Hsu NY Ilnytska O Belov G Santiana M Chen YH Takvorian PM. Viral reorganization of the secretory pathway generates distinct organelles for RNA replication. Cell. (2010) 141:799–811. 10.1016/j.cell.2010.03.050 20510927 PMC2982146

[12] Paul D Hoppe S Saher G Krijnse-Locker J Bartenschlager R. Morphological and biochemical characterization of the membranous hepatitis C virus replication compartment. J Virol. (2013) 87:10612–27. 10.1128/JVI.01370-13 23885072 PMC3807400

[13] Martin-Acebes MA Vazquez-Calvo Á Caridi F Saiz JC Sobrino F “Lipid involvement in viral infections: present and future perspectives for the design of antiviral strategies,” In: Baez RV editor. Lipid Metabolism. (2013). p. 291–322. 10.5772/2928

[14] Heaton NS Randall G. Multifaceted roles for lipids in viral infection. Trends Microbiol. (2011) 19:368–75. 10.1016/j.tim.2011.03.007 21530270 PMC3130080

[15] Wang Y Qian Y Fang Q Zhong P Li W Wang L. Author correction: saturated palmitic acid induces myocardial inflammatory injuries through direct binding to TLR4 accessory protein MD2. Nat Commun. (2018) 9:16185. 10.1038/ncomms16185 29553572 PMC5859348

[16] Sekar S Wu X Friis T Crawford R Prasadam I Xiao Y. Saturated fatty acids promote chondrocyte matrix remodeling through reprogramming of autophagy pathways. Nutrition. (2018) 54:144–52. 10.1016/j.nut.2018.02.018 29852453

[17] Librán-Pérez M Pereiro P Figueras A Novoa B. Antiviral activity of palmitic acid via autophagic flux inhibition in zebrafish (*Danio rerio*). Fish Shellfish Immunol. (2019) 95:595–605. 10.1016/j.fsi.2019.10.055 31676430

[18] Xu D Wang J Guo C Peng XX Li H. Elevated biosynthesis of palmitic acid is required for zebrafish against *Edwardsiella tarda* infection. Fish Shellfish Immunol. (2019) 92:508–18. 10.1016/j.fsi.2019.06.041 31247319

[19] Miyake T Hiasa Y Hirooka M Tokumoto Y Watanabe T Furukawa S. High serum palmitic acid is associated with low antiviral effects of interferon-based therapy for hepatitis C virus. Lipids. (2012) 47:1053–62. 10.1007/s11745-012-3716-8 22983804

[20] Hegde A Chen CL Qin QW Lam TJ Sin YM Characterization, pathogenicity and neutralization studies of a nervous necrosis virus isolated from grouper, *Epinephelus tauvina*, in Singapore. Aquaculture. (2002) 213:55–72. 10.1016/S0044-8486(02)00092-3

[21] Kara HM Chaoui L Derbal F Zaidi R de Boisséson C Baud M. Betanodavirus-associated mortalities of adult wild groupers *Epinephelus marginatus* (Lowe) and *Epinephelus costae* (Steindachner) in Algeria. J Fish Dis. (2014) 37:273–8. 10.1111/jfd.12020 24397531

[22] Qin QW Chang SF Ngoh-Lim GH Gibson-Kueh S Shi C Lam TJ. Characterization of a novel ranavirus isolated from grouper *Epinephelus tauvina*. Dis Aquat Organ. (2003) 53:1–9. 10.3354/dao053001 12608562

[23] Yu M Ren C Qiu J Luo P Zhu R Zhao Z. Draft genome sequence of the opportunistic marine pathogen vibrio harveyi strain E385. Genome Announc. (2013) 1:e00677-13. 10.1128/genomeA.00677-13 24336361 PMC3861414

[24] Huang YH Yu YP Yang Y Yang M Zhou LL Huang XH. Antiviral function of grouper MDA5 against iridovirus and nodavirus. Fish Shellfish Immunol. (2016) 54:188–96. 10.1016/j.fsi.2016.04.001 27050314

[25] Huang YH Yu YP Yang YY Yang M Zhou LL Huang XH. Fish TRIM8 exerts antiviral roles through regulation of the proinflammatory factors and interferon signaling. Fish Shellfish Immunol. (2016) 54:435–44. 10.1016/j.fsi.2016.04.138 27150052 PMC7130058

[26] Wang Q Yang M Li C Wang SW Wang YX Lin FM. Functional analysis of the CXCR1a gene response to SGIV viral infection in grouper. Fish Shellfish Immunol. (2019) 88:217–24. 10.1016/j.fsi.2019.02.046 30807858

[27] Yu YP Huang YH Wei SN Li PF Zhou LL Ni SW. A tumour necrosis factor receptor-like protein encoded by Singapore grouper iridovirus modulates cell proliferation, apoptosis and viral replication. J Gen Virol. (2016) 97:756–66. 10.1099/jgv.0.000379 26691529 PMC5381394

[28] Yu YP Huang XH Zhang JC Liu JX Hu Y Yang Y. Fish TRIM16L exerts negative regulation on antiviral immune response against grouper iridoviruses. Fish Shellfish Immunol. (2016) 59:256–67. 10.1016/j.fsi.2016.10.044 27815200

[29] Yu YP Huang YH Yang Y Wang SW Yang M Huang XH. Negative regulation of the antiviral response by grouper LGP2 against fish viruses. Fish Shellfish Immunol. (2016) 56:358–66. 10.1016/j.fsi.2016.07.015 27436518

[30] Yu YP Huang XH Liu JX Zhang JC Hu Y Yang Y. Fish TRIM32 functions as a critical antiviral molecule against iridovirus and nodavirus. Fish Shellfish Immunol. (2017) 60:33–43. 10.1016/j.fsi.2016.11.036 27847343

[31] Yu YP Huang YH Ni SW Zhou LL Liu JX Zhang JC. Singapore grouper iridovirus (SGIV) TNFR homolog VP51 functions as a virulence factor via modulating host inflammation response. Virology. (2017) 511:280–9. 10.1016/j.virol.2017.06.025 28689858

[32] Zhang Y Wang LQ Huang XH Wang SW Huang YH Qin QW Fish cholesterol 25-hydroxylase inhibits virus replication via regulating interferon immune response or affecting virus entry. Front Immunol. (2019) 10:322 10.3389/fimmu.2019.00322 30894855 PMC6414437

[33] Huang XH Huang YH Sun JJ Han X Qin QW Characterization of two grouper *Epinephelus akaara* cell lines: application to studies of Singapore grouper iridovirus (SGIV) propagation and virus–host interaction. Aquaculture. (2009) 292:172–9. 10.1016/j.aquaculture.2009.04.019

[34] Qin QW Lam TJ Sin YM Shen H Chang SF Ngoh GH. Electron microscopic observations of a marine fish iridovirus isolated from brown-spotted grouper, *Epinephelus tauvina*. J Virol Methods. (2001) 98:17–24. 10.1016/S0166-0934(01)00350-0 11543880

[35] Gómez-Lechón MJ Donato MT Martínez-Romero A Jiménez N Castell JV O'Connor JE. A human hepatocellular *in vitro* model to investigate steatosis. Chem Biol Interact. (2007) 165:106–16. 10.1016/j.cbi.2006.11.004 17188672

[36] Wang SW Huang XH Huang YH Hao X Xu H Cai M. Entry of a novel marine DNA virus, Singapore grouper iridovirus, into host cells occurs via clathrin-mediated endocytosis and macropinocytosis in a pH-dependent manner. J Virol. (2014) 88:13047–63. 10.1128/JVI.01744-14 25165116 PMC4249105

[37] Li C Liu JX Zhang X Yu YP Huang XH Wei JG. Red grouper nervous necrosis virus (RGNNV) induces autophagy to promote viral replication. Fish Shellfish Immunol. (2020) 98:908–16. 10.1016/j.fsi.2019.11.053 31770643

[38] Maier T Jenni S Ban N. Architecture of mammalian fatty acid synthase at 4.5 A resolution. Science. (2006) 311:1258–62. 10.1126/science.1123248 16513975

[39] Reed LJ Muench H A simple method of estimating 50% endpoints. Am J Epidemiol. (1938) 27:493–7. 10.1093/oxfordjournals.aje.a118408

[40] Diaz G Melis M Batetta B Angius F Falchi AM. Hydrophobic characterization of intracellular lipids *in situ* by Nile Red red/yellow emission ratio. Micron. (2008) 39:819–24. 10.1016/j.micron.2008.01.001 18329888

[41] Greenspan P Mayer EP Fowler SD. Nile red: a selective fluorescent stain for intracellular lipid droplets. J Cell Biol. (1985) 100:965–73. 10.1083/jcb.100.3.965 3972906 PMC2113505

[42] Yao HR Liu J Plumeri D Cao YB He T Lin L. Lipotoxicity in HepG2 cells triggered by free fatty acids. Am J Transl Res. (2011) 3:284–91. 21654881 PMC3102573

[43] Huang YH Huang XH Cai J OuYang ZL Wei SN Wei JG. Identification of orange-spotted grouper (*Epinephelus coioides*) interferon regulatory factor 3 involved in antiviral immune response against fish RNA virus. Fish Shellfish Immunol. (2015) 42:345–52. 10.1016/j.fsi.2014.11.025 25463297

[44] Tisoncik-Go J Gasper DJ Kyle JE Eisfeld AJ Selinger C Hatta M. Integrated omics analysis of pathogenic host responses during pandemic H1N1 influenza virus infection: the crucial role of lipid metabolism. Cell Host Microbe. (2016) 19:254–66. 10.1016/j.chom.2016.01.002 26867183 PMC5271177

[45] Ventura R Mordec K Waszczuk J Wang Z Lai J Fridlib M. Inhibition of *de novo* palmitate synthesis by fatty acid synthase induces apoptosis in tumor cells by remodeling cell membranes, inhibiting signaling pathways, and reprogramming gene expression. EBioMedicine. (2015) 2:808–24. 10.1016/j.ebiom.2015.06.020 26425687 PMC4563160

[46] Muñoz G Ovilo C Noguera JL Sánchez A Rodríguez C Silió L. Assignment of the fatty acid synthase (FASN) gene to pig chromosome 12 by physical and linkage mapping. Anim Genet. (2003) 34:234–5. 10.1046/j.1365-2052.2003.00987.x 12755829

[47] Portovedo M Ignacio-Souza LM Bombassaro B Coope A Reginato A Razolli DS. Saturated fatty acids modulate autophagy's proteins in the hypothalamus. PLoS ONE. (2015) 10:e0119850. 10.1371/journal.pone.0119850 25786112 PMC4364755

[48] Mizushima N Yoshimori T Levine B. Methods in mammalian autophagy research. Cell. (2010) 140:313–26. 10.1016/j.cell.2010.01.028 20144757 PMC2852113

[49] Yu X Li R Shi W Jiang T Wang Y Li C. Silencing of MicroRNA-21 confers the sensitivity to tamoxifen and fulvestrant by enhancing autophagic cell death through inhibition of the PI3K-AKT-mTOR pathway in breast cancer cells. Biomed Pharmacother. (2016) 77:37–44. 10.1016/j.biopha.2015.11.005 26796263

[50] Lorizate M Kräusslich HG. Role of lipids in virus replication. Cold Spring Harb Perspect Biol. (2011) 3:a004820. 10.1101/cshperspect.a004820 21628428 PMC3179339

[51] Martín-Acebes MA Blázquez AB Jiménez de Oya N Escribano-Romero E Saiz JC. West Nile virus replication requires fatty acid synthesis but is independent on phosphatidylinositol-4-phosphate lipids. PLoS ONE. (2011) 6:e24970. 10.1371/journal.pone.0024970 21949814 PMC3176790

[52] Nchoutmboube JA Viktorova EG Scott AJ Ford LA Pei Z Watkins PA. Increased long chain acyl-Coa synthetase activity and fatty acid import is linked to membrane synthesis for development of picornavirus replication organelles. PLoS Pathog. (2013) 9:e1003401. 10.1371/journal.ppat.1003401 23762027 PMC3675155

[53] García-Sastre A. Lessons from lipids in the fight against influenza. Cell. (2013) 154:22–3. 10.1016/j.cell.2013.06.024 23827671

[54] Dittharot K Jittorntam P Wilairat P Sobhonslidsuk A. Urinary metabolomic profiling in chronic hepatitis B viral infection using gas chromatography/mass spectrometry. Asian Pac J Cancer Prev. (2018) 19:741–8. 10.22034/APJCP.2018.19.3.741 29582629 PMC5980850

[55] Sun X Song L Feng S Li L Yu H Wang Q. Fatty acid metabolism is associated with disease severity after H7N9 infection. EBioMedicine. (2018) 33:218–29. 10.1016/j.ebiom.2018.06.019 29941340 PMC6085509

[56] Hsieh YC Chen YM Li CY Chang YH Liang SY Lin SY. To complete its replication cycle, a shrimp virus changes the population of long chain fatty acids during infection via the PI3K-Akt-mTOR-HIF1α pathway. Dev Comp Immunol. (2015) 53:85–95. 10.1016/j.dci.2015.06.001 26112000

[57] Huang YH Zhang Y Zheng JY Wang LQ Qin QW Huang XH Metabolic profiles of fish nodavirus infection *in vitro:* RGNNV induced and exploited cellular fatty acid synthesis for virus infection. Cell Microbiol. (2020) 11:790 10.1111/cmi.13216 32388899

[58] Kwan HY Fu X Liu B Chao X Chan CL Cao H. Subcutaneous adipocytes promote melanoma cell growth by activating the Akt signaling pathway: role of palmitic acid. J Biol Chem. (2014) 289:30525–37. 10.1074/jbc.M114.593210 25228694 PMC4215233

[59] Wang Z Liu D Zhang Q Wang J Zhan J Xian X. Palmitic acid affects proliferation and differentiation of neural stem cells *in vitro*. J Neurosci Res. (2014) 92:574–86. 10.1002/jnr.23342 24446229

[60] Wang X Liu JZ Hu JX Wu H Li YL Chen HL. ROS-activated p38 MAPK/ERK-Akt cascade plays a central role in palmitic acid-stimulated hepatocyte proliferation. Free Radic Biol Med. (2011) 51:539–51. 10.1016/j.freeradbiomed.2011.04.019 21620957

[61] Izdebska M Piatkowska-Chmiel I Korolczuk A Herbet M Gawronska-Grzywacz M Gieroba R. The beneficial effects of resveratrol on steatosis and mitochondrial oxidative stress in HepG2 cells. Can J Physiol Pharmacol. (2017) 95:1442–53. 10.1139/cjpp-2016-0561 28759727

[62] Gunduz F Aboulnasr FM Chandra PK Hazari S Poat B Baker DP. Free fatty acids induce ER stress and block antiviral activity of interferon alpha against hepatitis C virus in cell culture. Virol J. (2012) 9:143. 10.1186/1743-422X-9-143 22863531 PMC3490746

[63] Hu Y Huang YH Liu JX Zhang JC Qin QW Huang XH. TBK1 from orange-spotted grouper exerts antiviral activity against fish viruses and regulates interferon response. Fish Shellfish Immunol. (2018) 73:92–9. 10.1016/j.fsi.2017.12.010 29222027

[64] Jiang XS Chen XM Wan JM Gui HB Ruan XZ Du XG. Autophagy protects against palmitic acid-induced apoptosis in podocytes *in vitro*. Sci Rep. (2017) 7:42764. 10.1038/srep42764 28225005 PMC5320537

[65] Yu L Alva A Su H Dutt P Freundt E Welsh S. Regulation of an ATG7-beclin 1 program of autophagic cell death by caspase-8. Science. (2004) 304:1500–2. 10.1126/science.1096645 15131264

[66] Huang YH Huang XH Gui JF Zhang QY. Mitochondrion-mediated apoptosis induced by *Rana grylio* virus infection in fish cells. Apoptosis. (2007) 12:1569–77. 10.1007/s10495-007-0089-1 17551838

[67] Clarke P Tyler KL. Apoptosis in animal models of virus-induced disease. Nat Rev Microbiol. (2009) 7:144–55. 10.1038/nrmicro2071 19148180 PMC2772826

[68] Liang Q Luo Z Zeng J Chen W Foo SS Lee SA. Zika virus NS4A and NS4B proteins deregulate Akt-mTOR signaling in human fetal neural stem cells to inhibit neurogenesis and induce autophagy. Cell Stem Cell. (2016) 19:663–71. 10.1016/j.stem.2016.07.019 27524440 PMC5144538

[69] Lin Y Wu C Wang X Liu S Zhao K Kemper T. Glucosamine promotes hepatitis B virus replication through its dual effects in suppressing autophagic degradation and inhibiting MTORC1 signaling. Autophagy. (2020) 16:548–61. 10.1080/15548627.2019.1632104 31204557 PMC6999643

[70] Moscat J Diaz-Meco MT. p62 at the crossroads of autophagy, apoptosis, and cancer. Cell. (2009) 137:1001–4. 10.1016/j.cell.2009.05.023 19524504 PMC3971861

[71] Manley S Williams JA Ding WX. Role of p62/SQSTM1 in liver physiology and pathogenesis. Exp Biol Med. (2013) 238:525–38. 10.1177/1535370213489446 23856904 PMC4096157

[72] Komatsu M Kageyama S Ichimura Y. p62/SQSTM1/A170: physiology and pathology. Pharmacol Res. (2012) 66:457–62. 10.1016/j.phrs.2012.07.004 22841931

[73] Komatsu M Ichimura Y. Physiological significance of selective degradation of p62 by autophagy. FEBS Lett. (2010) 584:1374–8. 10.1016/j.febslet.2010.02.017 20153326

[74] Yoshii SR Mizushima N. Monitoring and measuring autophagy. Int J Mol Sci. (2017) 18:1865. 10.3390/ijms18091865 28846632 PMC5618514

[75] Boya P González-Polo RA Casares N Perfettini JL Dessen P Larochette N. Inhibition of macroautophagy triggers apoptosis. Mol Cell Biol. (2005) 25:1025–40. 10.1128/MCB.25.3.1025-1040.2005 15657430 PMC543994

[76] Jia K Yuan Y Liu W Liu L Qin Q Yi M. Identification of inhibitory compounds against Singapore grouper iridovirus infection by cell viability-based screening assay and droplet digital PCR. Mar Biotechnol (NY). (2018) 20:35–44. 10.1007/s10126-017-9785-1 29209860

